# Brazilian Primary and Secondary Public Oral Health Attention: Are Dentists Ready to Face the COVID-19 Pandemic?

**DOI:** 10.1017/dmp.2020.342

**Published:** 2020-09-10

**Authors:** Anya P.G.F. Vieira-Meyer, Maíra B. Coutinho, Helena P.G. Santos, Maria V. Saintrain, George T. de M. Candeiro

**Affiliations:** Family Health Program, Oswaldo Cruz Foundation – Ceará (Fiocruz-CE), Eusébio, Brazil; Faculty of Dentistry, Christus University Center (Unichristus), Fortaleza, Brazil; Municipal Health Secretary, Fortaleza City Hall – CE, Fortaleza, Brazil; Public Health Program, University of Fortaleza, Fortaleza, Brazil

**Keywords:** COVID-19 pandemic, dentistry, international health, primary health care, public health

## Abstract

**Objective:**

To investigate knowledge and practice of Brazilian public primary and secondary health care dentists during the coronavirus disease (COVID-19) pandemic.

**Methods:**

An online questionnaire with sociodemographic and COVID-19 knowledge questions was used.

**Results:**

A total of 4048 dentists working in the Brazilian public primary and secondary health care system were investigated; 4024 (99.41%) believe that COVID-19 can be transmitted through dental procedures. A fair level of COVID-19 symptoms knowledge by these dentists was observed (3.76±1.27 of 6.00), as well as the skepticism in personal protective equipment (3382; 83.55%) and biosafety procedures (3278; 80.98%) used as an efficient form of COVID-19 transmission prevention. Country region, performance of social distancing, dental specialty, the use of personal protective equipment, and biosafety preventive measures influenced the likelihood of dentists to perform dental treatment, either elective or urgent, during the COVID-19 pandemic.

**Conclusion:**

The need of extra preventive barriers for dental treatment may bring an extra financial stress in the Brazilian public primary and secondary health care system, as well as in the patient-dentist relationship, which may have to be reframed. Internationally accepted public guideline policies regarding dental treatment safety, as well as the technological development of preventive tools, are needed to deal with the challenges brought by COVID-19.

## INTRODUCTION

The 2019 coronavirus disease (COVID-19) is a viral infection caused by severe acute respiratory syndrome coronavirus 2 (SARS-CoV-2). It was first reported in Wuhan, China, in 2019, and has since spread around the world with exponential growth. Currently, it affects individuals on all continents, in one of the largest pandemics in history.^1^ The World Health Organization declared it a pandemic on March 11, 2020.^2^

Its symptoms can range from a common cold, with fever, malaise, stuffy nose and dry cough, to more severe findings, involving dyspnea and short of breath, as seen in Middle East respiratory syndrome (MERS) and severe acute respiratory syndrome (SARS).^3-5^ Other symptoms are headache, myalgia, sore throat, diarrhea, sputum production, hemoptysis, and vomiting.^4-8^ These symptoms can vary in magnitude among infected people, and some may test positive for the virus being asymptomatic, whereas others may have mild, medium, or severe symptoms.^6,8^ About 1 to 2% of the cases culminate in death.^3^

The transmission routes for SARS-CoV-2 include direct ways, such as droplet inhalation through coughing, sneezing, or even speech; and contact transmission, by oral, nasal, and eye mucous membranes.^9^ It has been confirmed that asymptomatic individuals are capable of spreading the virus.^10^ Saliva is one of the body fluids used for COVID-19 tests.^11^ The biggest concern with COVID-19 is the speed with which the disease spreads, contaminating many people at the same period. In this scenario, the health systems may be challenged.^12^

As it is a novel disease, there is neither an established treatment protocol nor vaccine for its prevention.^3^ Hence, several countries have adopted self-isolation or social/physical distancing as an effective measure to reduce the rates of mass transmission and to prevent their health care systems from collapsing.^13^

Currently, the social distancing has been recommended by the government and is being practiced in most cities of Brazil. However, professionals from essential areas must continue working. Among these are health care professionals, including dentists.^2^ The Brazilian Unified Health System (SUS), a public (free of charge) universal system for oral health, is divided into 3 strata – primary (primary care to oral health), secondary (specialized oral care), and tertiary (oral care in hospitals).^14^ Primary health care is organized by the family health team, composed of a doctor, a nurse, a dentist, health assistants, and community health agents. The secondary level of care is met by specialty dental clinics. During the pandemic, all SUS levels are working in a coordinate manner to prevent the COVID-19 spread and care for those in need (either COVID-19-infected individuals or those with other health problems, such as oral health emergencies).

Therefore, we sought to understand the levels of knowledge and practice of Brazilian dentists, from primary and secondary public health care, during the COVID-19 pandemic in Brazil.

## METHODS

**FIGURE 1:**
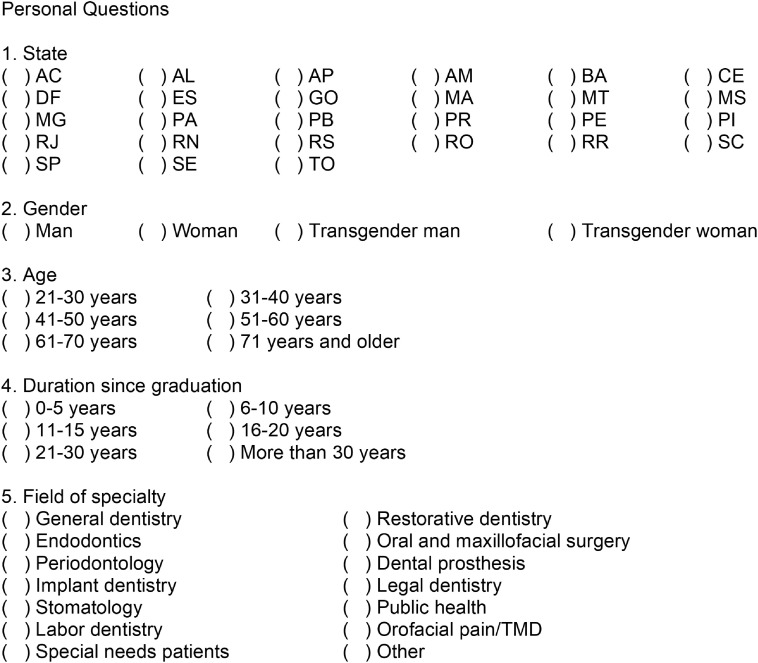
Part 1 (Personal Questions) of Online Questionnaire, Created in Google Forms (Google LLC, Mountain View, CA), Used to Collect Data on Sociodemographic Questions From Dentists Working in the Brazilian Primary and Secondary Health Care Systems (2020).

**FIGURE 2:**
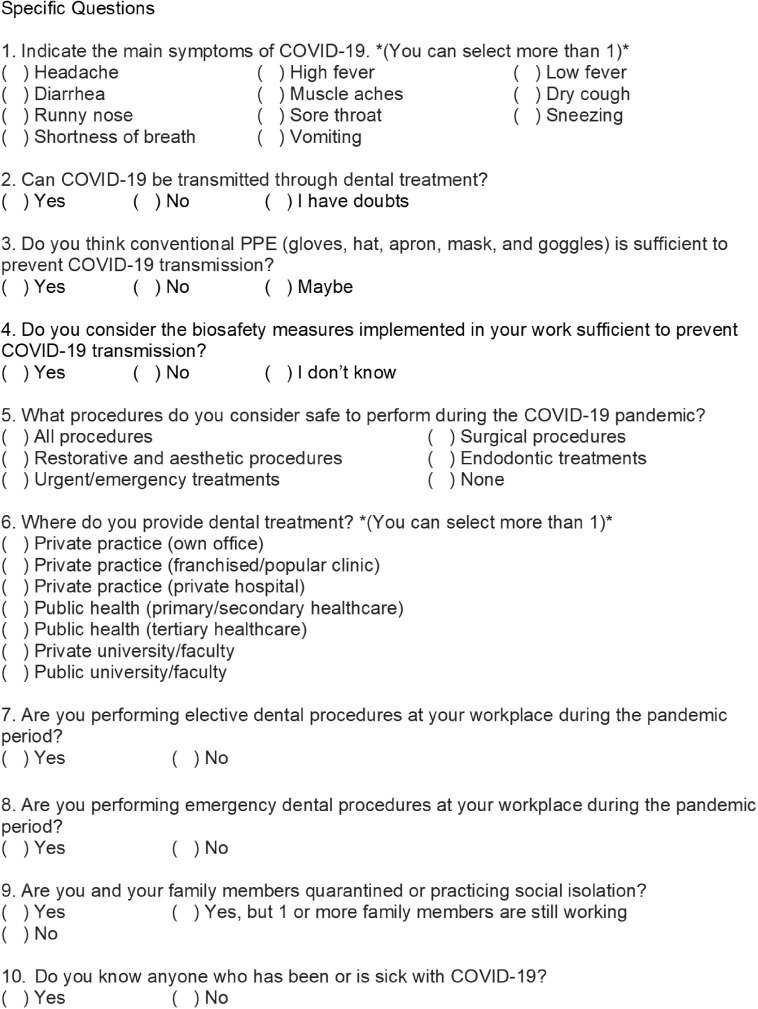
Part 2 (Specific Questions) of Online Questionnaire, Created in Google Forms (Google LLC, Mountain View, CA), Used to Collect Data on COVID-19 Transmission, Symptoms and Biosafety Care From Dentists Working in the Brazilian Primary and Secondary Health Care Systems (2020).

An online questionnaire was created using Google Forms (Google LLC, Mountain View, CA) to be answered by Brazilian dentists. It included questions (Figures 1 and 2) on sociodemographics and knowledge about COVID-19 transmission, symptoms, and related biosafety care. This questionnaire was sent to professionals through a link, via networking media – WhatsApp, Instagram, and Facebook. The data collection lasted for 7 days – March 29 to April 4, 2020. Using a sample-size calculator (https://www.surveysystem.com/sscalc.htm), the required sample size of 384 was estimated based on the number of dentists registered in the Brazilian Federal Dental Council (N = 336 700), 95% confidence level and 5% confidence interval.

Descriptive and bivariate analyses were performed. The data were analyzed using Stata SE 14.2 (StataCorp, College Station, TX). The mean and standard deviation were calculated for continuous variables, while frequencies and percentages were calculated for categorical data. The main symptoms (headache, high fever, diarrhea, muscle pain, dry cough, and shortness of breath) were considered to evaluate the dentists’ knowledge of COVID-19. The number of correct answers was counted for each dentist. Statistical tests, such as chi-square, t-test, and 1-way ANOVA, were used for bivariate analysis. Statistical significance was set at *P* < 0.05. This research was approved by the Ethics Committee of Christus University (Unichristus), Brazil (Protocol number: 30535020.5.0000.5049).

## RESULTS

Of the 16 578 dentists who filled out the questionnaire, 4048 stated that they work in primary or secondary public health care and were evaluated in this study. Of those, 4024 (99.41%) believed that COVID-19 can be transmitted through dental procedures; 3382 (83.55%) did not believe that the personal protective equipment (PPE) they routinely use is enough to protect from SARS-CoV-2 contamination; and 3278 (80.98%) consider the biosafety measures usually used in the day-to-day dental care insufficient to prevent COVID-19 contamination.

Some of the professionals (n = 1140; 28.16%) had not performed any type of procedure during the social distancing period, whereas 2908 (71.84%) are attending to patients, primarily emergencies. Out of these, 239 (5.91%) were still carrying out elective procedures. As for social distancing, 1638 (40.46%) stated that they were doing it. One quarter (25.59%) of the professionals know someone who tested positive for COVID-19.

Table 1 describes the knowledge of the evaluated dentists regarding the symptoms associated with COVID-19, whereas Table 2 describes the other information presented in the questionnaire.

**TABLE 1:** COVID-19 Symptoms* Knowledge Among 4024 Dentists of the Primary or Secondary Public Health Care System in Brazil – Absolute and Percentage Values (2020)

COVID-19 Symptoms	Yes	No
Headache	1835 (45.33%)	2213 (54.67%)
High fever	3269 (80.76%)	779 (19.24%)
Low fever	939 (23.20%)	3109 (76.80%)
Diarrhea	1295 (31.99%)	2753 (68.01%)
Muscle pain	1306 (32.26%)	2742 (67.74%)
Cough	3744 (92.49%)	304 (7.51%)
Running nose	1575 (38.91%)	2473 (61.09%)
Sore throat	2565 (63.36%)	1483 (36.64%)
Sneeze	979 (24.18%)	3069 (75.82%)
Shortness of breath	3760 (92.89%)	288 (7.11%)
Vomiting	271 (6.69%)	3777 (93.31%)

*Symptoms commonly reported in the literature.^3-8^

**TABLE 2:** Sociodemographic Information of 4024 Dentists of the Primary or Secondary Public Health Care System in Brazil, As Well As Their Knowledge on COVID-19 Transmission, and Biosafety Practice During COVID-19 Pandemic (2020)

Variable	Frequency (n)	Percentage (%)
**Country region**		
North	168	4.15%
Northeast	1163	28.73%
Southeast	2023	49.98%
South	333	8.23%
Midwest	361	8.92%
**Age**		
21-30 years old	679	16.77%
31-40 years old	1038	25.64%
41-50 years old	1309	32.34%
51-60 years old	866	21.39%
61-70 years old	151	3.73%
More than 70 years old	5	0.12%
**Gender**		
Female	2776	68.58%
Male	1270	31.37%
Male transgenic	1	0.02%
Female transgenic	1	0.02%
**Years since dental degree**		
0-5 years	639	15.79%
6-10 years	431	10.65%
11-15 years	528	13.04%
16-20 years	663	16.38%
21-30 years	1155	28.53%
More than 30 years	632	15.61%
**Dental specialty**		
General practitioner	1165	28.78%
Buco-maxilo-facial surgeon	112	2.77%
Restorative dentistry	127	3.14%
Orofacial pain/TMD	34	0.84%
Endodontics	551	13.61%
Stomatology	36	0.89%
Implantology	251	6.20%
Labor dentistry	24	0.59%
Legal dentistry	25	0.62%
Special needs patients	74	1.83%
Periodontology	202	4.99%
Prosthesis	236	5.83%
Public health	462	11.41%
Other	749	18.50%
**Dental work location besides primary/secondary public health**		
Private practice	2137	52.79%
Private practice in low cost health unit	329	8.13%
Hospital private practice	59	1.46%
Hospital public practice	118	2.92%
Private universities/faculties practice	118	2.92%
Public universities/faculties practice	40	0.99%
**Performed procedures**		
None	2531	62.52%
Aesthetic restoration	60	1.48%
Surgeries	76	1.88%
Endodontic treatment	75	1.85%
Emergency treatment	1405	34.71%
All procedures	136	3.36%
**COVID-19 can be transmitted by dental treatment**		
Yes	4024	99.41%
No	5	0.12%
I´m in doubt	19	0.47%
**Personal protective equipment (PPE) sufficient to prevent COVID-19 transmission**		
Yes	232	5.73%
No	3382	83.55%
Maybe	434	10.72%
**Your actual biosecurity measures are sufficient to prevent COVID-19 infection**		
Yes	451	11.14%
No	3278	80.98%
Don´t know	319	7.88%
**Performing elective procedures at your workplace**		
Yes	239	5.91%
No	3804	94.09%
**Performing urgent procedures at your work place**		
Yes	2908	71.84%
No	1140	28.16%
**Social distancing**		
Yes	1638	40.46%
No	375	9.26%
Yes, but some of my family members are still working	2035	50.27%
**Do you know someone with COVID-19**		
Yes	1036	25.59%
No	3012	74.41%

As mentioned earlier, the main symptoms (headache, high fever, diarrhea, muscle pain, dry cough, and shortness of breath) were considered to evaluate the knowledge about COVID-19 among the dentist respondents. The number of correct answers was counted for each dentist, where 6 was the maximum score. The average found was 3.76(±1.27).

There was found to be no statistical difference in COVID-19 knowledge among different age groups (*P* = 0.4707). Upon analyzing the time since graduation, it was observed that professionals who graduated recently (0-5 years) were poorly informed as compared with the other groups (*P* = 0.0485). When stratified by gender, female dentists seemed to be better informed about the disease than males (*P* = 0.0006).

In geographical aspects, there was no difference on COVID-19 symptoms among the 5 different regions of Brazil (*P* = 0.1389), nor among groups that believed or not that the virus could be transmitted through dental care (*P* = 0.6214). Interestingly, dentists who considered that PPE habitually used by them was sufficient to prevent COVID-19 contamination demonstrated lesser knowledge about COVID-19 symptoms when compared with their counterparts (*P* = 0.007). Similar results were observed among groups that disagreed on the protective ability of the biosafety protocols. Moreover, those who were unsure about the protection provided by biosafety protocols were found to have less expertise regarding the symptoms (*P* = 0.0013).

There was no difference in the knowledge of professionals, whether or not they were performing elective (*P* = 0.7608) or emergency (*P* = 0.5799) procedures during the pandemic. With respect to social isolation, professionals with more knowledge were found to be the ones who were not complying with social isolation guidelines (*P* = 0.0148). Of those who were practicing social isolation, 57.45% were attending to emergency procedures, while this value was 88.80% for those who were not in social isolation (*P* < 0.05). Only 4.65% of those in social isolation were performing elective treatments against 8.27% of those not in social isolation (*P* = 0.008).

Of the dentists who believed that SARS-CoV-2 could be transmitted during dental care, only 5.8% were undertaking elective treatments. On the other hand, 40% of those who did not believe in this form of spread continued to perform dental procedures (*P* < 0.05).

With regard to elective and urgent care, professionals who believed that routinely used PPE is sufficient for their protection showed a tendency to perform more urgent treatments (*P* = 0.055) and, certainly, more elective treatments (*P* < 0.05) when compared with those who discredited PPE. Dentists (79.82%) who believed that the usual biosafety procedures are adequate for COVID-19 prevention were performing emergency dental treatments, whereas this value was 70.84% among dentists who did not believe the same (*P* < 0.05). In terms of elective procedures, 11.11% of the dentists who considered the current available biosafety protocols as sufficient were performing them, whereas this value was 4.86% among those who did not believe the same (*P* < 0.05). Those who are most performing urgent procedures are specialists in surgery, stomatology, legal dentistry, and public health (*P* = 0.001).

Some regional differences were also observed. Dentists in the South (79.28%), mid-West (78.12%), and Southwest (75.23%) regions were found to be performing urgent dental treatment more commonly as compared with those in the North (66.07%) and Northeast (62.68%) regions (*P* < 0.05). In cases of elective procedures, the North (10.71%), South (7.21%), and Southeast (6.14%) regions displayed the highest percentages of elective treatment, whereas the mid-West (5.82%) and Northeast (4.48%) regions displayed the lowest (*P* = 0.014). Meanwhile, dentists in the North (44.05%), Northeast (40.41%), and Southeast (41.52%) regions seemed to be practicing social isolation better as compared with those in the South (39.04%) and mid-West (34.35%) regions (*P* < 0.005).

The dentists evaluated in this study included only those who were working in the primary and secondary public health care sectors. However, most often, these professionals often also have other workplaces. There was no difference in the knowledge between dentists who did or did not have secondary jobs in private practices (*P* = 0.9397), popular clinics (*P* = 0.7482), private hospitals (*P* = 0.5832), public hospitals (*p* = 0.3274), and private universities (*P* = 0.9214). However, dentists who also worked at public universities demonstrated a significantly higher level of knowledge (*P* = 0.0369).

## DISCUSSION

The relevance of this article is related to the proportion taken by the COVID-19 pandemic and at the greater risk of infection by the dental personnel.^15^ The results revealed that at least 3 of the main symptoms associated with COVID-19 (dry cough, shortness of breath, and high fever) were well-known to dentists in the primary and secondary levels of care. However, other symptoms, such as headache, muscle pain, and sore throat were comparatively lesser known to them. Our results also showed no relationship between the knowledge of COVID-19 symptoms and execution of dental treatment, either urgent or elective, during the pandemic. It is not clear whether there is a relation between this knowledge and safer treatment care during the COVID-19 pandemic. However, in the case of other viral infections, such as hepatitis B, hepatitis C, and HIV/AIDS, it has been demonstrated that dentists’ level of knowledge of a disease plays a particularly important role in forming their attitudes and practices regarding patients.^16^

The infection of salivary glands by coronavirus is being given increasing importance and attention in recent studies, in which the potential diagnosis of COVID-19 in saliva is revealed,^17^ as well as its connection with the development of asymptomatic infections.^18^ Ren et al.^19^ reported that loss of taste is an early symptom of COVID-19. The fact that this symptom appears before fever and other common symptoms of COVID-19 supports the hypothesis that the oral cavity, especially the mucosa of the tongue, may be an initial site of infection for SARS-CoV-2.^19^ According to The *New York Times,* dentists are at the greatest risk of infection,^20^ since they work directly with salivary fluids in the form of aerosols, which have a great potential for viral transmission.^21^ This places the dentist community, especially the ones in primary health care, in an extremely vulnerable position, as they are the frontline oral health care professionals caring for patients, some of whom may be asymptomatic. This calls for greater preparation, including more protective barriers for the safety of these individuals while they perform their work.

Almost all dentists who participated in this study agreed that COVID-19 can be transmitted during dental treatment. However, the results on the biosecurity measures highlight an important concern. While dentists who considered their PPE and biosafety procedures to be sufficient in preventing COVID-19 infection were more prone to perform urgent and elective dental care, a high percentage of professionals believed that the current biosecurity measures and routinely used PPE are not sufficient to prevent COVID-19 infection. It is not clear whether this high percentage is due to a low level of preventive methods being used in primary and secondary dental care, or due to the professionals’ lack of knowledge regarding biosecurity and clinically safe practices during the pandemic. Most likely, they did not have access to protocols and reliable guidelines to improve their knowledge in this area. Despite not being universally accepted, there are reliable research articles describing preventive measures in the dental setting.^10,22^ These articles describe in detail some practical strategies to block virus transmission, such as patient evaluation, hand hygiene, personal protective measures, rinsing the mouth before any dental procedures, rubber dam isolation, disinfection of the clinic settings, and management of medical waste. However, the scientific information does not seem to be flowing to all dentists at the desired speed. It remains in the personal interest to seek for most of this information, and the scientific method is probably not dominated by most professionals. Therefore, it is important to have a leadership in the dissemination of adequate information during a pandemic, as the overload and the uncertainties caused by confused and contradictory articles may disorient rather than clarify the biosecurity and PPE methods that must be used.^23^ Public policies are extremely important in the dissemination of proper information. On that manner, SUS has played an important role in responding to the disease, with initiatives to improve its capacity and with the establishment of specific protocols for the clinical management of patients, and a good control on the community transmission of COVID-19.^24^ However, a protocol for dental treatment is yet to be established. Additionally, as the primary health care system is run by the municipalities, some differences may be seen as the implementation of COVID-19 preventive measures in different parts of the country, which is also influenced by the financial challenges faced, and the availability of supplies. Additionally, as Brazil is a highly populated country with an extended continental territory, individual state governors are enforcing social distancing measures based on the number of cases and intensive care unit occupancy rates in public and private hospitals.^25^ Differences in the rules of social isolation among the various regions of the country may be influencing social isolation/distancing and the provision of elective and urgent care to some extent.

A study carried out among Jordanian dentists working in various levels of care corroborates with our findings and the findings of another study.^23,26^ It establishes that dentists are aware of COVID-19 symptoms, mode of transmission, infection controls, and measures in dental clinics. However, many dental practices lack the minimum requirements for infection control, despite the availability of prevention guidelines.

We observed an evident incompatibility between knowledge of COVID-19 symptoms and social isolation/distancing. One would assume that the higher the knowledge, higher would be the adherence to social isolation/distancing. However, the present study revealed that this was not true. This can largely be attributed to the fact that dental professionals working in public primary and secondary health care services are considered essential and, therefore, cannot stop working. Generally, in Brazil, dentists undergo exams to enter the public service in Brazil, which may reflect this higher knowledge among those not maintaining social distancing. Nevertheless, this is only a supposition, as the data collected are not sufficient to test this hypothesis.

An important finding of the present study is that a considerable percentage of the study population was carrying out dental procedures other than emergencies, despite not believing that the biosafety care protocols were sufficient. This poses a real risk of cross-infection and COVID-19 dissemination, especially since there is not yet a universal protocol or guideline available regarding dental care for active or suspected COVID-19 cases.^27^ Nevertheless, as occurred with other viral infections, such as HIV/AIDS, international accepted guidelines/protocols, as well as the technological development of preventive tools, need to be prepared to deal with the COVID-19 challenges.

Professionals who graduated recently (0-5 years) were more poorly informed on COVID-19 symptoms than the other groups. This finding corroborates with the study of Rostamzadeh et al., which deals with other viral infections and obtained a lower level of knowledge in recent graduates.^21^ The higher knowledge may be due to clinical experience or postgraduate courses. This is further supported by a recent study on dentists’ knowledge of COVID-19, which found that professionals with higher qualifications (postgraduates) reported better knowledge scores as compared with recent graduates.^28^

It is unavoidable to discuss that post-pandemic, the cost of dental treatment will probably increase. New treatment protocols in the patient-dentist relationship might also be needed, especially in the case of pediatric and special-needs patients. The SUS is financed by the public government, and, despite its importance in providing health care to the Brazilian population, it has been suffering from insufficient funding for a long time.^29^ After the acute COVID-19 pandemic, the need for implementation of additional preventive measures during dental treatment will add to the financial stress on primary public health service and may interfere with the amount and diversity of treatments offered. Management of pediatric and special-needs patients quite often demand close contact with the dentist. Commonly, this involves interactions without dental masks and PPE before the dental treatment itself, in order to familiarize the patient with the dental environment and personnel, which is fundamental in the patient-dentist bond and allows for less traumatic and better quality treatment. The additional preventive barriers, which are necessary to prevent the COVID-19 infection, might interfere with this interaction and, therefore, make dental treatment complex.

## CONCLUSION

The present study showed a fair level of knowledge of COVID-19 symptoms among dentists working in the primary (family health program) and secondary health care system. It also revealed that many of them do not believe that PPE and biosafety procedures used in their dental practices are efficient in preventing and protecting against COVID-19 transmission. Nevertheless, we found that the region in the country, practice of social isolation, belief in PPE and biosafety preventive measures influenced the likelihood of dentists performing elective or urgent dental treatment during the COVID-19 pandemic period. After the acute COVID-19 pandemic period, the need for extra preventive barriers during dental treatment may lead to extra financial stress on the primary and secondary health care system, as well as on the patient-dentist relationship, guidelines for which may have to be reframed. Thus, new internationally accepted public guideline policies regarding dental treatment safety will need to be developed along with technologically advanced preventive tools to deal with the challenges posed by the novel coronavirus.
